# Is Fasting Good When One Is at Risk of Liver Cancer?

**DOI:** 10.3390/cancers14205084

**Published:** 2022-10-17

**Authors:** Iulia Minciuna, Laurens A. van Kleef, Horia Stefanescu, Bogdan Procopet

**Affiliations:** 1 Regional Institute of Gastroenterology and Hepatology Octavian Fodor, 400394 Cluj-Napoca, Romania; 2 3rd Medical Department, University of Medicine and Pharmacy Iuliu Hatieganu, 400347 Cluj-Napoca, Romania; 3 Department of Gastroenterology and Hepatology, Erasmus MC University Medical Centre, 3015 GD Rotterdam, The Netherlands

**Keywords:** intermittent fasting, time-restricted feeding, hepatocellular carcinoma, non-alcoholic fatty liver disease, cirrhosis

## Abstract

**Simple Summary:**

Intermittent fasting has the potential to beneficially act upon several pathways that are involved in hepato-cellular carcinoma pathogenesis. Especially among non-alcoholic steatohepatitis patients (NASH), intermittent fasting could effectively reduce the chance of progression to advanced liver disease and hepatocellular carcinoma. Although intermittent fasting activates similar pathways among those without NASH, the altered metabolism in cirrhotic patients may make them exceptionally vulnerable to malnutrition and increase the risk of liver-related adverse events.

**Abstract:**

Hepatocellular carcinoma (HCC), one of the leading causes of cancer-related deaths worldwide, is a multistep process that usually develops in the background of cirrhosis, but also in a non-cirrhotic state in patients with non-alcoholic fatty liver disease (NAFLD) or viral hepatis. Emerging evidence suggests that intermittent fasting can reduce the risk of cancer development and could improve response and tolerance to treatment through the metabolic and hormonal adaptations induced by the low energy availability that finally impairs cancer cells’ adaptability, survival and growth. The current review will outline the beneficial effects of fasting in NAFLD/NASH patients and the possible mechanisms that can prevent HCC development, including circadian clock re-synchronization, with a special focus on the possibility of applying this dietary intervention to cirrhotic patients.

## 1. Introduction

Hepatocellular carcinoma (HCC) accounts for 90% of primary liver cancers, and represents the fourth leading cause of cancer-related death worldwide [1]. Although HCC arises in the background of cirrhosis, in 20% of cases, it can develop in a non-cirrhotic state, with chronic viral hepatitis B or C and non-alcoholic fatty liver disease (NAFLD) accounting for most of these cases [2,3].

Present day lifestyles are characterized by increased sedentary behavior and easy access to and increased intake of high-caloric food. These lifestyles have led to the worrisome prevalence of obesity, diabetes and NAFLD, which are risk factors for hepatic and extra-hepatic cancer. However, increased caloric intake is not the only determining factor for deteriorating metabolic health, since the timing of food intake is also a key player in maintaining the body’s metabolic flexibility. Intermittent fasting, in which timing of food intake is more important than the quantity, may therefore have a range of potential benefits. Indeed, pre-clinical and clinical studies have outlined the beneficial effects of intermittent fasting (IF) in establishing weight loss, improving cardio-metabolic health, preventing neurodegenerative disease, reducing the risk of cancer development and prolonging lifespan [4,5,6].

Intermittent fasting encompasses various eating patterns and is characterized by alternating periods of eating and fasting that vary in duration from 12 up to 24 h for two to seven days a week. The most-studied regimens of intermittent fasting include (i) alternate day fasting (ADF); (ii) 5:2 intermittent fasting (fasting for two non-consecutive days a week); (iii) time-restricted feeding (restricting the eating period to a specific daily timeframe that usually ranges from 6 to 8 h) and maybe the oldest form of intermittent fasting is considered (iv) Ramadan fasting, when people abstain from food and liquids from sunrise to sunset, with fasting duration ranging from 12 to 18 h depending on the geographical area [7].

We will further review the evidence characterizing the interphase between liver cancer and fasting metabolism, with an additional focus on populations at risk that would most benefit. We included data from animal and human studies that included an IF diet intervention, namely alternate day fasting, 5:2 diet, time-restricted feeding or Ramadan fasting.

## 2. Fasting and Cirrhosis

Hepatocellular carcinoma arises primarily in a cirrhotic state; therefore, the population at risk of developing HCC is represented by cirrhotic patients. However, applying a restrictive dietary intervention to a group of patients at risk of malnutrition and sarcopenia may have serious adverse effects that may not outweigh the potential benefits.

Malnutrition is common in cirrhotic patients and is attributed to decreased oral intake, maldigestion and malabsorption, which have multifactorial origins [8,9]. Decreased oral intake is due to anorexia, dysgeusia and sodium-restricted unpalatable diets, while malabsorption can be secondary to a decreased luminal bile acid concentration, to portal hypertensive enteropathy and intestinal dysbiosis [9,10]. However, malnutrition in cirrhotic patients is primarily determined by metabolic alterations characterized by an accelerated state of starvation, with an early shift from glucose to lipid utilization as the primary energy source during the post-absorptive state [11,12]. This early use of lipids is further aggravated by the diminished liver glycogen stores [12]. In these patients, glucose metabolism dysfunction is also outlined by glucose intolerance or insulin resistance, which seem to be present in up to 70% of cirrhotic patients, while 14–46% of them have type II diabetes mellitus [13].

Among cirrhotic patients that fast overnight, 75% of the total energy metabolism is from lipids, which is reflected by increased rates of ketogenesis and reduced respiratory quotation and is equivalent to the metabolic state of 2–3 days of fasting in healthy individuals [14,15]. Moreover, as the rate of gluconeogenesis increases during periods of starvation, there is an increase in amino acid utilization as an energy source, which further accelerates skeletal muscle proteolysis, reduces protein synthesis and leads to sarcopenia [16].

Apart from age, sex and ethnicity, the severity and etiology of liver disease can impact skeletal muscle mass, with alcoholic and cholestatic liver disease being associated with the most severe muscle mass loss. Skeletal muscle mass is the balance between muscle protein synthesis (MPS) and muscle protein breakdown (MPB) that alternate in the fed (postprandial) and fasted (post-absorptive) state. Food-entrained insulin release can indirectly regulate MPB, with the highest rates of MPB being achieved in the post-absorptive state characterized by low insulin levels, to provide free amino acids (AA) that can be stored as proteins in skeletal muscles or other organs or be used as gluconeogenic precursors.

Dietary amino acids are the primary precursors for synthesizing new skeletal muscle proteins. After the consumption of an equivalent dose of approximately 0.25 g/kg of leucine-enriched dietary protein during a single meal, there is achieved a saturating dose of AA for the postprandial stimulation of MPS [17,18]. Moreover, after achieving the peak MPS, usually 1.5–3 h after the protein feeding, MPS returns gradually to the baseline values even in the presence of a sustained increase in plasma aminoacidemia, a phenomenon referred to as “muscle full” effect [19,20,21,22]. This outlines the presence of a refractory period following protein ingestion, with the MPS pathway not being able to be stimulated sequentially for a period of 3–5 h [22]. A possible mechanism to prolong the postprandial muscle protein synthetic response is resistance training, with actually a similar maximal stimulatory protein dose to the one at rest (0.3 g/kg) [17].

Introducing an intermittent fasting dietary intervention may actually have a deleterious effect on muscle mass in cirrhotic patients, due to the hypercatabolic effect of fasting and the anabolic resistance described in this population. This effect is likely to correlate strongly with the intake restriction duration, with the worst results for ADF or the 5:2 diet. Still, applying TRF with an adjusted protein intake of approximately 1.6 g/kg divided into feeding windows separated by 3–5 h, with the addition of resistive training, would theoretically favor MPS and preserve muscle mass in cirrhotic patients [16].

Significant increases in serum bilirubin level, prothrombin time and decreases in albumin level were demonstrated by a few studies evaluating the effect of Ramadan fasting in cirrhotic patients. Strikingly, up to 41% developed ascites during and 64% after Ramadan, and 13% of patients progressed to Child Pugh Class C during and 32.6% after Ramadan fasting [23]. Contrastingly, another prospective multicenter study showed decreased BMI, glucose, AST, ALT, GGT and ALP levels predominantly in male patients with Child Pugh Class A without previous history of gastrointestinal bleeding, while older patients with diabetes mellitus and with Child Pugh Class C developed bilirubin elevation during the fasting period [24]. Table 1 illustrates the identified studies reporting on outcomes and safety of Ramadan fasting in cirrhotic patients.

Aligning with the global pandemic, obesity is also highly prevalent among patients with end-stage liver disease as illustrated by the 33% obesity among US patients undergoing liver transplantation [27]. Importantly, the presence of obesity gives an approximately two-fold increased risk of HCC development, four-fold increased HCC-related risk of mortality and a two-fold increased risk of life-threatening complications [28,29,30]. Apart from patients with NAFLD, obesity is also frequently encountered in patients with other etiologies such as chronic hepatitis B or C and alcohol-related liver disease [31].

Patients with end-stage liver disease and obesity have an increased risk of hepatic decompensation independent of portal pressure and liver function, increased risk of infections, acute-on-chronic liver failure and HCC development [31,32]. Moreover, cirrhotic patients with obesity can have an additional loss of muscle mass, leading to the development of sarcopenic obesity, a condition associated with a poorer prognosis [33].

Obesity may favor the development of HCC through the increased hepatic oxidative stress and inflammation secondary to altered microbiome composition and altered white adipose tissue adipokine production, namely increased levels of leptin and decreased levels of adiponectin [34,35,36]. Moreover, the increase in substrate availability that characterizes obesity favors the chronic activation of insulin and insulin-like growth factor (IGF) receptors, which results in increased cell glucose uptake, cell proliferation and angiogenesis, creating a pro-malignant microenvironment [36].

Therefore, in obese cirrhotic patients without features of sarcopenic obesity, dietary interventions such as intermittent fasting can reduce hepatic and systemic inflammation and insulin resistance, re-balance the levels of adipokines and reduce the risk of liver decompensation, finally decreasing the risk of HCC development.

## 3. Fasting in NASH Patients

In accordance with the previously mentioned obesity pandemic, NASH is also increasingly prevalent and one of the main drivers for advanced chronic liver disease in the general population.

Intermittent fasting may especially be beneficial in NASH patients. In this population, HCC can arise in a non-cirrhotic state, skipping the step of cirrhosis that precedes HCC development in most chronic liver disease etiologies. NASH is a multifactorial disease, characterized by excess hepatic fat and increased inflammation, and is often the result of a sedentary lifestyle and/or high caloric food intake that result in increased substrate availability, comprising primarily carbohydrates and fatty acids. These substrates contribute to the expansion of hepatic glycogen storage and liver triglyceride formation. Other important factors that may contribute to NASH are microbiota dysbiosis, metabolic dysfunction and genetic predisposition [37,38,39]. Intermittent fasting can act on many of the main pathogenetic chains of NASH, and may therefore alleviate its phenotype and prevent disease progression and carcinogenesis.

### 3.1. Metabolic Flexibility

Metabolic flexibility reflects the body’s ability to coordinate substrate sensing, trafficking, deposit and utilization as well as to choose the adequate metabolic pathway according to fuel need and availability to maintain energy availability [40,41,42]. As set forth, in NASH this flexibility is lost, leading to a metabolic inflexibility characterized by inadequate substrate utilization that results in dyslipidemia, insulin resistance and hepatic fat accumulation [40]. Intermittent fasting may restore metabolic flexibility by resynchronizing the circadian clock and acting upon the main metabolic drivers involved in NASH pathogenesis. During fasting, in order to maintain energy homeostasis, there is a shift from energy-consuming processes (such as cell growth, protein and glycogen synthesis) to processes that stimulate ATP production via FA oxidation and glucose uptake.

PPARs are key players of metabolism during fasting, favoring the upregulation of enzymes involved in FAs uptake and β oxidation, and inhibiting SREBP-mediated cholesterol and triglyceride synthesis [39,40]. The inverse correlation between PPARα gene expression and NASH histological severity could be responsible for NASH’s impaired FA oxidation and decreased level of ketone bodies, another possible key target of IF [41].

Insulin resistance is important in NASH pathogenesis, and leads to reduced glucose uptake in skeletal muscles and adipose tissue together with increased hepatic de novo lipogenesis and increased flux of free fatty acids [39]. Moreover, insulin resistance can induce adipose tissue dysfunction by altering the production of adipokines and inflammatory cytokines that can spill into hepatic circulation [43]. Moreover, insulin resistance leads to compensatory hyperinsulinemia, which upregulates the hepatic growth hormone (GH) receptor leading to increased hepatic release of IGF1 and exerting growth factor-like activity on hepatocytes [44,45]. Therefore, hyperinsulinemia increases the risk of liver cancer as it favors cell proliferation and inhibits apoptosis and also as it activates phosphatidylinositol 3-kinase (P13K)/protein kinase B (Akt) and mitogen-activated protein kinase (MAPK) by binding to the insulin receptor substrate (IRS) [46,47].

Moreover, fasting can also improve adipokine secretion by increasing adiponectin level and decreasing leptin level, which leads to a reduced inflammatory milieu and improved insulin sensitivity [48]. As fasting-induced lipolysis in adipose tissue results in a large reduction in adipocyte size, insulin sensitivity can be further enhanced due to the increased number of insulin receptors on the surface of adipocytes [49].

### 3.2. Inflammation

Although excessive lipids and lipopolysaccharides can determine hepatocyte production of certain chemokines and pro-inflammatory cytokines (TNFα, IL-6 and IL-1β), it is the injured hepatocytes that lead to the production of damage-associated molecular patterns (DAMPs) that can contribute to the inflammatory milieu by activating Kupffer cells [50,51,52]. Kupffer cells express TLR4 and the binding of lipopolysaccharides (LPS) leads to the activation of NF-κB, MAPK, ERK1, p38 and JNK. Intermittent fasting has an additional anti-inflammatory effect through the activation of PPARs, AMPK and SIRT1 pathways [53,54,55,56,57,58]. 

### 3.3. Autophagy and Oxidative Stress

Hepatic accumulation of free fatty acids, free cholesterol and other lipid metabolites leads to mitochondrial dysfunction with oxidative stress and endoplasmic reticulum stress-inducing hepatocyte injury and apoptosis [59,60]. Moreover, the increased level of FFA and triglycerides suppresses the initiation of autophagy through the suppression of serine/threonine-protein kinase ULK1 activity and the activation of mTOR, further contributing to the increase in oxidative stress. The oxidative damage, combined with the presence of chronic inflammation and the hepatic proliferative response, can promote fibrosis and carcinogenesis [61]. Intermittent fasting stimulates autophagy and mitophagy through AMPK and PPAR inhibitory effects upon mTOR, and the AMPK activating effect upon FOXO. Increased autophagy promotes the clearance of oxidatively damaged mitochondria and misfolded or unfolded proteins generated under ER stress conditions, which are used as prime materials for anabolic processes in times of low nutrient availability [62].

### 3.4. Gut Microbiota

Intermittent fasting may also beneficially affect the gut microbiota. Changes in gut microbiota among NASH patients alter intestinal immunity and disrupt small bowel permeability favoring the translocation of bacteria or bacterial products with conserved motifs (PAMPs) that are recognized by pathogen recognition receptors of various hepatic cell types, finally leading to hepatic inflammation and fibrosis [63]. Intermittent fasting can improve the composition and function of gut microbiota, aiding replication and repair mechanisms of the gut lining by promoting an increase in mucus secretion during the night. Intermittent fasting may thereby prevent bacterial translocation and lipopolysaccharide-induced endotoxemia and reverse the changes seen in NASH patients [64].

### 3.5. Adverse Events

Although in the cirrhotic population IF should be applied with caution in highly selected cases, most clinical studies performed in non-cirrhotic patients have outlined that an IF diet as alternate day fasting or time-restricted feeding can cause non-severe adverse events [65]. They consist mostly of mild headaches, nausea, dizziness, constipation, intermittent visual disorders and episodes of hypoglycemia, especially in diabetic patients using sulfonylureas and/or insulin that need close monitoring and dose adjustment during the fasting intervention [65,66,67,68,69]. While few case reports have highlighted the potentially deleterious effect of fasting-induced ketoacidosis in patients with type I or II diabetes mellitus, the additional favoring factors consisting in the association of a ketogenic diet or fasting for several consecutive days may have also contributed to this effect [70,71]. Moreover, several articles analyzing the incidence of diabetic ketoacidosis during Ramadan month did not find an increase in comparison to non-fasting months [72,73,74].

In summary, intermittent fasting may act upon several important pathways in NASH patients, including improvements in metabolic flexibility, gut microbiota and inflammation. Clinical studies have shown, apart from a significant decrease in body weight, that intermittent fasting has additional cardiometabolic benefits comprising in improvements in blood pressure, LDL cholesterol, insulin resistance, inflammation and oxidative stress with mild adverse events [75,76,77,78]. Through these improvements, intermittent fasting may be beneficial in the regression of NASH, as well as the prevention of advanced liver disease, including fibrosis and hepatocellular carcinoma.

## 4. Fasting and Cancer

The low glucose levels during fasting stimulate pancreatic glucagon secretion and favor glucose synthesis through glycogenolysis and gluconeogenesis. After liver glycogen depletion, there is a switch from glucose to lipid metabolism, with a preferential utilization of fatty acid-derived ketones in spite of glucose by cells with high metabolic activity such as skeletal muscles and neurons [79]. Moreover, prolonged fasting determines an increase in serum level of catecholamines and corticosteroids that favors white adipose tissue lipolysis with the subsequent release of increased levels of free fatty acids into the bloodstream as a primary source for β oxidation [79]. Apart from being an energy source, ketone bodies also have important signaling functions, including the activation of transcription factor cyclic AMP response element–binding protein (CREB) and regulating the expression and activity of peroxisome proliferator–activated receptor γ coactivator 1α (PGC-1α), fibroblast growth factor and sirtuins [80]. Moreover, ketone bodies such as β-hydroxybutyrate can act as an endogenous histone deacetylase inhibitor, thus protecting against oxidative stress and slowing tumor development [4].

The increased level of FFAs can also lead to the activation of transcription factors such as peroxisome proliferator–activated receptor α (PPAR-α), inducing an increase in FGF21 production [81,82].

Due to its interaction with 5′ AMP-activated protein kinase (AMPK), mammalian target of rapamycin (mTOR) and Sirtuin (SIRT) pathways, PPAR is one of the main drivers of metabolism during fasting, promoting fatty acid β-oxidation in the liver, skeletal muscle and adipose tissue.

**Figure 1:**
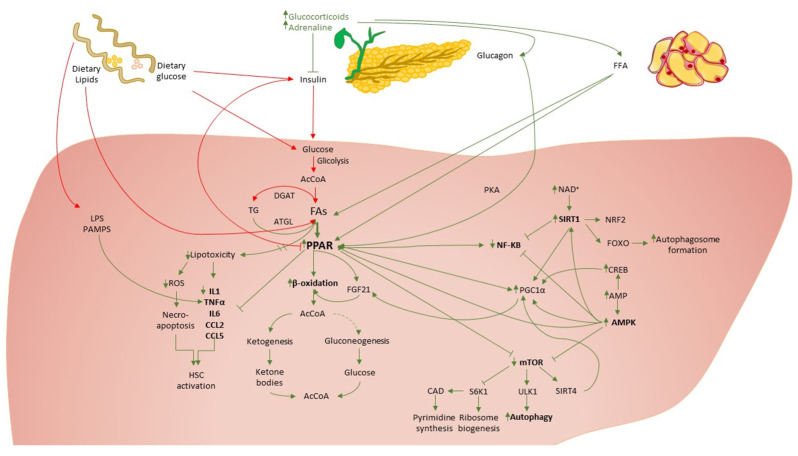
The molecular mechanisms involved in fasting response. LPS: lypopolysacharides,: reactive oxygen species, IL1: interleukine 1, TNFα: tumor necrosis factor α, CCL2, 5: C-C Motif Chemokine Ligand 2, 5, TG: triglycerides, DGAT: diacylglycerol acyltransferase, FAs: fatty acids, FFA: free fatty acids, PPAR: peroxisome proliferator-activated receptor alpha, FGF21: Fibroblast growth factor 21, AcCoA: Acetyl-coenzyme A, NF-κB: nuclear factor kappa B, FOXO: Forkhead box O, CREB: cAMP responsive element binding protein, AMPK: 5′ AMP-activated protein kinase, SIRT1: Sirtuin 1, Bhlhb2: basic helix-loop-helix family member, NAD: Nicotinamide adenine dinucleotide, AMP: Adenosine monophosphate, NRF2: NF-E2–related factor 2, mTOR: mammalian target of rapamycin, PGC1α: peroxisome proliferator-activated receptor gamma coactivator 1-alpha, ULK1: Unc-51 Like Autophagy Activating Kinase 1, S6K1: S6 kinase beta-1, CAD: carbamoyl-phosphate synthetase 2, aspartate transcarbamoylase, dihydroorotase.

The low energy intake from periods of fasting induces changes in the ratios of bioenergetic sensors NAD+ to NADH, ATP to AMP and acetyl CoA to CoA [6]. These changes lead to the activation of downstream proteins, including kinases such as AMPK, deacetylases such as SIRT1 and transcription factors such as FOXOs, PGC-1α and NRF2 [6]. These downstream proteins inhibit anabolic processes, favor lipid catabolism, reduce inflammation, activate autophagy and induce mitochondrial biogenesis. Moreover, the decreased circulating level of amino acids, the downregulation of the insulin–insulin-like growth factor 1 (IGF-1) signaling pathway and the inhibitory effect of the upregulated PPARα and AMPK pathways suppress the activity of mTOR, thus further stimulating autophagy, reducing protein synthesis and cell growth (Figure 1) [83,84,85].

The interaction between HCC pathogenic chains and the pathways involved in the fasting response is intricate and dependent on the moment of applying intermittent fasting in the natural history of HCC development.

The potential anti-carcinogenic effect of fasting can also be derived from studies outlining that drugs whose actions mimic fasting effects can inhibit hypoxia-induced metastasis, angiogenesis and metabolic reprogramming in HCC, as is the case with SGLT2 inhibitors [86,87].

So far, numerous signaling pathways involved in HCC carcinogenesis that have been identifiedcan be grossly grouped into five main categories, with emerging pathways still under research: (1) tyrosine kinase-dependent growth factor receptors and their downstream mediators, (2) pathways involved in differentiation and cell-cell signaling, (3) inflammation pathways, (4) epigenetic pathways and (5) angiogenesis [88,89].

### 4.1. Insulin Growth Factor and HCC

The insulin growth factor signaling system regulates cell proliferation and growth and inhibits apoptosis in times of protein and nutrient availability [90,91]. Insulin growth factor deregulations identified to play a role in HCC development consist in IGF-1R overexpression, IGF2 overexpression, IGFBP3 downregulation and allelic loss of IGF2R [92]. IGF-1R can further induce the phosphorylation of β-catenin and can favor E-cadherin dissociation, leading to weaker cell-cell adhesions [93]. Moreover, elevated levels of IGF1 and insulin can activate phosphatidylinositol-3-kinase (PI3K)/AKT/mTOR and Ras/Mitogen-activated protein kinase (MAPK) pathways that are involved in HCC pathogenesis [94].

The PI3K/AKT/mTOR pathway favors HCC development by promoting neovascularization via HIF-1α and vascular endothelial growth factor (VEGF) upregulated expression and by promoting tumor invasion, metastasis and cell cycle progression [95,96]. Insulin-mediated PI3K activation has a stimulatory effect upon mTORC1, leading to decreased autophagy and increased anabolic processes and cell proliferation [94]. mTORC1 is upregulated in up to 40–50% of human HCCs and favors the development of less differentiated tumors, leading to a poorer prognosis [97].

During fasting, FGF21 can lower IGF1 levels by inhibiting phosphorylated STAT5 in the liver [4]. In addition to a decrease in the circulating levels, in times of low nutrient availability there is also a reduction in IGF1 biological activity secondary to increased insulin-like growth factor-binding protein 1 levels that binds to circulating IGF1 and inhibits its interaction with the appropriate cell surface receptors [5].

By reducing the levels of glucose, insulin and IGF1, fasting can inhibit the PI3K/AKT/mTOR pathway that is known to be involved in HCC pathogenesis.

Mutations and epigenetic modifications that increase growth and promote insensitivity to anti-growth signals can make cancer cells vulnerable to fasting as they lose the ability to adapt to a variety of extreme environments [98]. By reducing glucose availability and increasing fatty acid β-oxidation, fasting promotes a switch from aerobic glycolysis (Warburg effect) that characterizes energy metabolism in cancer cells, to mitochondrial oxidative phosphorylation [5]. This switch leads to increased ROS production as a result of increased mitochondrial respiratory activity and also reduction in cellular redox potential owing to decreased glutathione synthesis from glycolysis and the pentose phosphate pathway [99]. The combined effect of ROS augmentation and reduced antioxidant protection boosts oxidative stress and leads to apoptosis in cancer cells.

### 4.2. Inflammation and HCC

The anti-inflammatory effect of fasting can also prevent the development and progression of liver cancer, as HCC is considered an inflammation-linked cancer with more than 90% of cases developing in the context of hepatic injury and inflammation [100].

The perpetuation of a non-resolving inflammatory response is characterized by immune cell infiltration, mainly tumor-associated macrophages, immature myeloid cells and T cells, by the dysregulated production of cytokines with the balance leaning towards pro- (TNF-α, IL-6, IL-1) rather than anti-inflammatory cytokine (IL-10, IL-12, TGFβ) production and by the occurrence of angiogenesis and tissue remodeling that favors the sequence fibrosis-cirrhosis-HCC [101].

Chronic inflammation results in the generation of ROS and reactive nitrogen species (NOS) that can lead to DNA strand breaks, single-base mutations, or can induce post-translational modification of proteins that control cell cycle or survival [102,103,104]. Inflammatory cytokines such as TNFα, IL-1β and TGFβ can determine ectopic expression of activation-induced cytidine deaminase (AID) which leads to mutations in HCC-associated oncogenes (TP53) or proto-oncogenes (MYC) [105]. Moreover, they can inhibit DNA repair and, in the case of IL6, promote mutated cell expansion leading to increased mutagenesis and genome instability [101].

One of the main drivers of the inflammatory response involved in HCC development is NF-κB transcription factor. NF-κB is activated in almost all chronic liver diseases and regulates pro-inflammatory, proliferative and pro-survival genes [106]. The NF-κB pathway can be activated canonically by pro-inflammatory cytokines such as TNFα and IL-1β or TLR agonists including PAMS and DAMPS and, non-canonically, by a small subset of cytokines that belong to the TNF family [107]. The NF-κB pathway can target genes encoding pro-inflammatory cytokines (TNF, IL-1 and IL-6), growth factors, chemokines, matrix metalloproteinases, pro-proliferative proteins, anti-apoptotic proteins, pro-inflammatory enzymes, angiogenic factors (such as VEGF) and adhesion molecules [108]. Moreover, NF-κB can polarize macrophages towards the M2 phenotype, which has tumor-promoter and immunosuppressive behavior. Thus, this shows that NF-κB is involved in cancer cell proliferation and survival, in the acquisition of cancer stem cell proprieties, invasion, angiogenesis and metastasis [108].

The anti-inflammatory effect of fasting is exerted through PPAR, Glucocorticoid receptor (GR), AMPK and SIRT1 activation. While PPAR α and PPARγ exert their anti-inflammatory effect by attenuating NF-κB-driven inflammatory cytokine and chemokine production, PPAR β/δ favors the suppression of pro-inflammatory adhesion molecules on vascular endothelial cells and drives Kupffer cells towards a more anti-inflammatory phenotype [53,109]. Moreover, fasting can induce GR activation that leads to a re-programming of the macrophage secretome, with suppressed secretion of TNF [110].

### 4.3. Autophagy and HCC

The role of autophagy in hepatocarcinogenesis is complex, as it has a dual effect consisting in a tumor suppressive function in the pre-malignant state while once the malignant phenotype has fully developed, autophagy can provide rapidly growing cancer cells with the necessary nutrients for survival.

Autophagy is a lysosome-dependent self-digestive process that aims to maintain cellular integrity in times of nutrient scarcity. During fasting, AMPK can activate autophagy via mTOR inhibition or by activating FOXO which is involved in the upregulation of several autophagy inducers [56]. SIRT1 is involved in both mitochondrial biogenesis and autophagy by deacetylating either PGC-1α at different lysine residues or autophagy regulators such as ATG5 and ATG7, while FGF21 interacts with PPARα to transcriptionally activate autophagy by upregulating TFEB and ATG7 gene expression [111,112].

Autophagy exerts its tumor-suppressive effects by modulating the inflammatory and immune response. Autophagy regulates development of lymphocytes, and their functional diversification as naïve T cell numbers is influenced by the presence of mitophagy while mature T cells rely on autophagy for their survival [113]. Moreover, lipid droplet accumulation in hepatocytes as a consequence of defective autophagy can generate the release of linoleic acid causing hepatic CD4+T cell depletion leading to an immunosuppressive pro-tumorigenic microenvironment [113]. Moreover, autophagy can stimulate effector T cells by decreasing the expression of PD-L1 by macrophages [114].

Autophagy exerts its anti-inflammatory effects by dampening inflammasome activation and type I IFN signaling and also by downregulating NF-κB signaling via NSFL1C cofactor p47 and by degrading BCL-10, as shown in antigen-activated T cells [115].

Still, autophagy can also have a tumor-promoter effect as it was shown that it can enhance tumor cell survival in the hypoxic regions of solid tumors and that in cell-expressing RAS oncogene, it is necessary for maintaining oxidative metabolism and for facilitating glycolysis [116].

### 4.4. Energy-Sensing Pathways and HCC

AMPK and SIRT1, sensors of intracellular energy, are also connecting chains between intermittent fasting and HCC pathogenesis.

AMPK is activated during fasting by increased levels of ADP to ATP ratio or AMP to ATP ratio. The first known function of AMPK is the regulation of lipid metabolism. AMPK inhibits de novo synthesis of FAs, cholesterol and triglycerides and activates FA uptake and β-oxidation [56]. In addition to the inhibition of lipid anabolism, AMPK activates lipid catabolism. AMPK increases FA uptake by controlling the translocation of FA transporter CD36 to the plasma membrane [56]. Protein synthesis is a high-energy process that is inhibited during energy stress to conserve cellular ATP. AMPK additionally inhibits mTORC1 activity via either phosphorylating tuberous sclerosis complex (TSC) 2 at Ser-1387 or by phosphorylating Raptor at Ser-792 [117].

In cirrhotic liver tissue, low levels of AMPK phosphorylation at Thr172 are associated with an increased incidence of HCC in comparison with those with high levels of AMPK phosphorylation [118]. Moreover, in HCC cells, AMPK activity is significantly decreased compared to non-tumoral liver tissue, a feature that further correlates with worse prognosis [119].

In tumor cells, AMPK signals cell cycle arrest, determines glucose starvation induced apoptosis and can affect cell motility, thus leading to a decreased metastatic capacity [85,120]. As it is downregulated in HCC, the AMPK pathway can exert its antitumoral effect during periods of fasting, when it is upregulated.

SIRT1, an intracellular energy sensor and a NAD-dependent deacetylase, is activated in times of fasting due to AMPK-induced fatty oxidation that increases the NAD+/NADH ratio. Moreover, SIRT1 negatively regulates GH-mediated IGF1 mRNA production and can shift FOXO-dependent responses from apoptosis to cell cycle arrest and stress resistance [121].

The role of SIRT1 in hepatic carcinogenesis is complex, multifaceted and still to be characterized. So far, studies have shown SIRT1 can have both a tumor-suppressor and a tumor-promoter effect depending on its predominant localization—cytoplasmatic versus nuclear—or on the cell type on which is overexpressed, normal hepatocytes versus HCC cells [83]. In normal hepatocytes, SIRT1 favors genomic stability and induces the mitochondrial unfolded protein response, leading to metabolic fitness and favoring cell viability [122]. In contrast, by interacting with PGC-1α, SIRT1 increases mitochondrial biogenesis, cellular ATP levels and DNA transcript levels, which boosts HCC metastasis [123,124]. Additionally, SIRT1 can exert HCC protective effects after binding and deacetylating β-catenin [83].

## 5. Circadian Clock and Liver Cancer

In order to adapt to the daily variation of food availability and the light-dark cycle, organisms have developed molecular clock mechanisms able to anticipate the changes and adjust and maintain metabolic homeostasis. The circadian clock, a self-sustained cell-autonomous oscillator present in every cell type, is entrained to an exact 24 h rhythm by environmental cues. It is regulated by zeitgebers, which are the time cues that phase shift its activity. Light is one of the most well characterized zeitgebers, which resets the central suprachiasmatic nucleus (SCN) clock via the retino-hypotalamic tract.

At the molecular level, the core of the circadian clock oscillator is represented by BMAL1 and CLOCK transcription factors that heterodimerize and activate genes containing E-box DNA binding sequences in their promoter enhancer regions, including a large number of clock-controlled genes (CCGs) but also Period (PER1, PER2), cryptochrome (CRY1, CRY2) genes and the nuclear hormone receptors Rev-Erb and ROR. In turn, PER1/2 and CRY1/2 proteins heterodimerize to inhibit the transcriptional activity of the BMAL/CLOCK complex, thus leading to the suppression of their own expression, while ROR stimulates and Rev-ERBs inhibit through ROR response element DBS the transcription of the activators BMAL1 and CLOCK [125,126,127,128,129]. These feedback loops generate circadian oscillations in the expression of approximately 20% of the genes that are involved in transcription, translation, signaling or in cellular processes such as cell cycle control, inflammation and metabolism [130].

The dysregulation of the circadian clock function can act on different levels in terms of energy balance, immune function, cell proliferation, DNA replication and repair, DNA damage response, apoptosis and senescence, leading to the development of metabolic disorders and cancers [131]. The dysregulation of the circadian clock function in different human cancers is closely associated with a constitutive activation of intracellular inflammatory and oncogenic signaling pathways including p38, c-Myc, NF-κB, BCL-XL, PKA, aberrant chromatic remodeling, deregulation of inflammatory cytokines and suppression of tumor suppressors ATM, p53 and p21 [132].

While a proper functioning of the circadian clock shows tumor-suppressing potential, the disturbance of circadian rhythmicity that is caused by, e.g., shift work in humans and chronic jet lag in animal models, represents an independent risk factor of HCC. In mouse models, chronic jet lag leads to the activation of the constitutive androstane receptor (CAR) which leads to β-catenin and c-Myc overexpression and favors hepatocarcinogenesis through the sequence NAFLD-NASH-cirrhosis/HCC [132,133].

The development and progression of HCC rely on complex molecular mechanisms involving genetic and epigenetic alterations to oncogenes and tumor suppressor genes and disturbed control and inappropriate interaction between important signaling pathways such as Wnt/β-catenin, Hedgehog, MAPK, NOTCH, JAK/STAT3 and PI3K/AKT/mTOR and the circadian clock that leads to metaflammation, disrupted cellular differentiation and growth, finally leading to carcinogenesis [134,135]. When compared to non-tumoral hepatic tissue, the evaluation of circadian genes and protein expression in HCC tissue reveals reduced expression levels of PER1, PER2, PER3 and CRY2 [136]. Moreover, PER2 and PER3 negatively correlate with tumor size [137]. A lack of PER2 increases c-Myc expression, leading to an increased susceptibility to HCC development after DEN injection while full deletion of CRY1 and CRY2 favors chemically-induced liver carcinogenesis [138,139].

Another contributing factor to HCC development is the disruption of circadian-controlled genes that have a hepato-protective function. Among these, adiponectin, which is under circadian clock control, prevents HCC development through hepatic activation of p38α and of AMPK, while FGF21 is essential for preventing NAFLD progression to HCC during a long-term obesogenic diet [140,141,142].

In physiological conditions, the circadian clock controls the production of cytokines and cytolytic factors, proliferation of leucocytes, activities of NK cells and redistribution of T and B lymphocytes, dendritic cells, leukocytes and macrophages to lymphoid organs. The disruption of circadian homeostasis favors the development of immunosuppression [143,144,145]. Ablation or deregulation of the core circadian genes Per1, Per2, Bmal1, Rev-erbα, or Clock in mice favors deregulation of pro-inflammatory cytokines, cytotoxic receptors, immunoregulatory genes, NK and mast cell activities, and inhibition of B lymphocyte differentiation [143,146,147,148,149,150]. The influence between the circadian clock and the immune system is bidirectional as the central pacemaker activity can be modulated by pro-inflammatory cytokines (IL-1 and IL-6), TNF-α and anti-inflammatory drugs that alter intracellular expression of Bmal1, Npas2, Cry1 and/or Per2 [132].

By restoring the diurnal rhythm, intermittent fasting holds the promise of reducing the risk of metabolic disease and cancer development.

## 6. Conclusions

By re-synchronizing the circadian clock and re-setting key metabolic pathways involved in cell proliferation, growth, defense and function, intermittent fasting is able to prevent the evolution of chronic liver disease towards liver cancer. In NASH patients, a population at risk of liver cancer even in a non-cirrhotic state, intermittent fasting can act upon most of the NASH pathogenic drivers, reducing not only the cancer risk but also the progression of the disease. Still, in patients with decompensated liver cirrhosis, intermittent fasting may have deleterious effects, further studies being needed to assess the effect of fasting in compensated cirrhotic patients and features of the metabolic syndrome that theoretically would mostly benefit.

## Figures and Tables

**Table 1:** The effects of Ramadan fasting in cirrhotic patients.

Author Year	Study Design	Study Population	Number of Patients	Results
Elnadry et al., 2011 [23]	Observational and comparative	Chronic hepatitis and Cirrhotics (Child A and B)	*N* = 202 Fasting: 103 patients (57 chronic hepatitis, 46 cirrhotics). Non-fasting: 99 patients (52 chronic hepatitis, 47 cirrhotics)	Dyspeptic symptoms were higher in fasting group Gastrointestinal bleeding during Ramadan was higher in fasting group compared to non-fasting, but the variceal bleeding was significantly higher in the non-fasting group Chronic hepatitis fasting group showed non-significant changes pre, during and post Ramadan regarding liver functions. In the fasting cirrhotic group, the decompensation to Child C class developed both during and after Ramadan fasting
Elfert et al., 2011 [24]	Observational and non-comparison	Cirrhotics (Child A, B and C)	*N* = 216	BMI, serum glucose, ALT, AST, GGT, and ALP decreased Serum bilirubin increased significantly after full Ramadan fast Male sex, Child Pugh Class A and absence of gastrointestinal bleeding bleeding were independent factors in reduction in liver enzymes and serum glucose during Ramadan fasting Older age, Diabetes mellitus status and Child Pugh Class C were independent factors for elevation of serum bilirubin and creatinine during Ramadan fasting Twenty seven patients discontinued their fast due to fatigue Variceal bleeding and encephalopathy reported in eight and six patients, respectively Ramadan fasting had no significant effect on PV diameter or portal blood flow
Mohamed et al, 2016 [25]	Observational and non-comparison	Cirrhotics (Child A, B and C)	*N* = 40	Cirrhotic patients showed significant short-term increases in the congestive index as a non-invasive marker of the portal blood flow After Ramadan, there was a statistically significant increase in bilirubin and a decrease in albumin in Child Pugh Class C In patients with Child Pugh Class A and B there was no significant change in bilirubin, albumin level, prothrombin concentration or the degree of ascites, encephalopathy or upper GI bleeding events before and after Ramadan Shifting towards more advanced stage of Child class was due to lower limb edema development, increased ascites, increased jaundice and development of overt hepatic encephalopathy In total, seven patients developed complications with two cases of variceal bleeding
Mohamed et al., 2018 [26]	Observational and comparative	Cirrhotic (Child A and B) and healthy volunteers	*N* = 72 Cirrhotic fasting (*N* = 34), Cirrhotic non-fasting (*n* = 8) Healthy volunteers fasting (*N* = 30)	Patients with cirrhosis showed changes in their portal hemodynamics with increased CI Although more increased in cirrhotic patients, the congestive index didn’t change when these patients fasted MELD score and serum albumin showed significant changes in comparison to healthy subjects but no differences between cirrhotic patients that fasted or not

BMI: body mass index, AST: aspartate aminotransferase, ALT: alanine transaminase, GGT: gamma-glutamyl transferase, ALP: alkaline phosphatase, CI: congestive index.

## Data Availability

Not applicable.
